# Oxidized LDL Downregulates ABCA1 Expression via MEK/ERK/LXR Pathway in INS-1 Cells

**DOI:** 10.3390/nu13093017

**Published:** 2021-08-29

**Authors:** Jingya Lyu, Kensaku Fukunaga, Hitomi Imachi, Seisuke Sato, Toshihiro Kobayashi, Takanobu Saheki, Tomohiro Ibata, Takafumi Yoshimura, Hisakazu Iwama, Koji Murao

**Affiliations:** 1 Department of Physiology, School of Medicine, Jinan University, 601 Huangpu Avenue West, Tianhe District, Guangzhou 510632, China; jingylyu@med.kagawa-u.ac.jp; 2 Department of Endocrinology and Metabolism, Faculty of Medicine, Kagawa University, 1750-1, Miki-cho, Kita-gun, Kagawa 761-0793, Japan; fukunaga@med.kagawa-u.ac.jp (K.F.); ihitomi@med.kagawa-u.ac.jp (H.I.); seisuke-310@med.kagawa-u.ac.jp (S.S.); koba1987@med.kagawa-u.ac.jp (T.K.); t-saheki@med.kagawa-u.ac.jp (T.S.); ibata@med.kagawa-u.ac.jp (T.I.); yoshimura.takafumi@kagawa-u.ac.jp (T.Y.); 3 Life Science Research Center, Kagawa University, 1750-1, Miki-cho, Kita-gun, Kagawa 761-0793, Japan; iwama@med.kagawa-u.ac.jp

**Keywords:** oxidized LDL, ABCA1, liver X receptor, MEK/ERK, insulin secretion, lipotoxicity

## Abstract

Impaired insulin secretion is one of the main causes of type 2 diabetes. Cholesterol accumulation-induced lipotoxicity contributes to impaired insulin secretion in pancreatic beta cells. However, the detailed mechanism in this process remains unclear. In this study, we proved that oxidized low-density lipoprotein (OxLDL) reduced insulin content, decreased PDX-1 expression, and impaired glucose-stimulated insulin secretion (GSIS) in INS-1 cells, which were rescued by addition of high-density lipoprotein (HDL). OxLDL receptors and cholesterol content were increased by OxLDL. Consistently, OxLDL suppressed cholesterol transporter ABCA1 expression and transcription in a dose-dependent and time-dependent manner. Inhibition of MEK by its specific inhibitor, PD98059, altered the effect of OxLDL on ABCA1 transcription and activation of ERK. Next, chromatin immunoprecipitation assay demonstrated that liver X receptor (LXR) could directly bind to ABCA1 promoter and this binding was inhibited by OxLDL. Furthermore, OxLDL decreased the nuclear LXR expression, which was prevented by HDL. LXR-enhanced ABCA1 transcription was suppressed by OxLDL, and the effect was cancelled by mutation of the LXR-binding sites. In summary, our study shows that OxLDL down-regulates ABCA1 expression by MEK/ERK/LXR pathway, leading to cholesterol accumulation in INS-1 cells, which may result in impaired insulin synthesis and GSIS.

## 1. Introduction

Diabetes mellitus is one of the most common chronic metabolic diseases and the ninth major cause of death. According to the report from Japan Diabetes Society, 1 in every 7 Japanese is suffering from diabetes and 95% of them are type 2 diabetes (T2D), which is characterized as impaired insulin secretion and insulin resistance [1]. The main cause for T2D is impaired insulin secretion from pancreatic beta cells, normally combined with abnormal lipid profiles, which exhibit higher concentration of plasma triglyceride and low-density lipoprotein (LDL)-cholesterol, as well as lower high-density lipoprotein (HDL)-cholesterol concentration [2]. Of note, a clinical report shows that the oxidatively-modified lipoprotein, oxidized LDL (OxLDL) has a positive relationship with T2D in 7 years of follow-up, suggesting that oxidative stress induced by OxLDL may be one novel risk factor of T2D [3].

A previous study demonstrated that OxLDL decreased the mRNA level of proinsulin and insulin secretion from HIT-T15 cells [4]. Following this study, insulin resistance and cholesterol accumulation were found in adipocytes treated with OxLDL [5,6]. Recent study showed that blocking OxLDL by its antibody significantly improved insulin sensitivity and reduced plasma level of cholesterol and triglyceride in obese Rhesus Macaques [7], pointing out the association between OxLDL and diabetes. Oxidized LDL is able to be recognized by lectin-type oxidized LDL receptor 1 (LOX-1) [8,9]. Uptake of OxLDL by its receptor induces cholesterol accumulation in macrophages and promotes formation of foam cells, leading to the atherosclerotic plaque formation [10,11]. Preventing the uptake of OxLDL by knockout of its receptor reduces atherosclerosis in mice [12].

Cholesterol accumulation in pancreatic beta cells results in cell apoptosis and impaired insulin secretion, which is called lipotoxicity of beta cells [13]. As a key regulator of reverse cholesterol transport, the ATP-binding cassette transporter A1 (ABCA1) is a 254-kD membrane protein to export lipid from cytoplasm to apolipoproteins in vivo [14]. A previous study showed that mice lacking of the pancreatic *ABCA1* gene exhibited defective glucose-stimulated insulin secretion (GSIS) and glucose intolerance [15]. In vitro, absence of ABCA1 impaired GSIS and altered cholesterol homeostasis in pancreatic islets [16]. Our previous study proved that downregulation of ABCA1 by angiotensin II increased cholesterol content and impaired GSIS in INS-1 cells and pancreatic islets [17]. This evidence pindicates that pancreatic ABCA1 expression is important to regulate the cholesterol content of beta cells and protect the function of beta cell in type 2 diabetes. Moreover, OxLDL has been demonstrated to reduce the expression of ABCA1 by inhibiting of liver X receptor (LXR) in HUVECs [18]. As a critical pathogenic marker of vascular atherosclerosis, there is accumulating evidence demonstrating that OxLDL increases cholesterol accumulation in macrophages and hepatocytes [19,20], which contributes to increase the prevalence of metabolic syndrome and fatty liver disease [20,21]. However, the effect of OxLDL on cholesterol accumulation in pancreatic beta cells is not clear. Additionally, the detailed mechanism of OxLDL-regulated pancreatic ABCA1 is not completely understood.

In this study, we checked the effect of OxLDL on the GSIS, cholesterol content mediated by ABCA1 expression in INS-1 cells, and hypothesized that OxLDL might induce cholesterol accumulation by downregulation of ABCA1 expression, then impaired function of pancreatic beta cells.

## 2. Materials and Methods

### 2.1. OxLDL and HDL

Oxidized LDL and HDL were purified from human plasma via ultracentrifugation to homogeneity as determined via agarose gel electrophoresis, which were purchased from BIO-RAD (5685-3557; 5685-2004).

### 2.2. Cell Culture

INS-1 cells, originally isolated from a rat insulinoma, were split every 7 days during 12~40 passages. These cells were maintained in RPMI1640 medium (Wako) supplemented with 10% heat-inactivated fetal bovine serum (FBS; Dainippon Pharmaceutical Co., Ltd., Tokyo, Japan), 50 μM beta-mercaptoethanol, 100 U/mL penicillin, and 100 μg/mL streptomycin. All cells were incubated in humidified 5% CO_2_ at 37 °C. When 80% confluent, the cells were starved with RPMI 1640 media containing 0.5% FBS for 6 h. After starvation, the cells were treated with varying doses of OxLDL or HDL for 24 h or varying time.

### 2.3. Western Blot Analysis

Proteins were separated by SDS-PAGE (7.5%) and transferred to polyvinylidene difluoride membrane for immunoblotting. After blocking with skim milk, the membrane was incubated with the 1st antibody for ABCA1, LXR, TFIID, Lox-1 (Santa Cruz Biotechnology Inc., Dallas, CA, USA), or phospho-MEK, MEK, phospho-ERK1/2, ERK1/2 (Cell Signal Technology, Tokyo, Japan) at 4 °C overnight or GAPDH antibody (Biomol Research, Plymouth Meeting, Pennsylvania, USA) at room temperature for 1 h [17]. The membrane was then incubated with the HRP-linked rabbit or mouse secondary antibody (DakoCytomation) at room temperature for 1 h. Protein bands were detected by ECL (GE Healthcare, Tokyo, Japan) under Luminescent image analyzer LAS-1000 Plus.

### 2.4. Reverse Transcription-Quantitative, Real-Time Reverse Transcriptase-Polymerase Chain Reaction

According to the manufacturer’s protocol, total RNA was extracted by the RNA-Bee reagent and 1 μg total RNA were synthesized with SuperScript II reverse transcriptase (Invitrogen). PCR was performed with LightCycler 480 SYBR Green Master (Roche) by using CFX96 Touch Real Time PCR Detection Systems (BIO-RAD). The sequences of the forward and reverse primers were referenced as previous studies [17,22] and shown as follows. *PDX-1*: 5′-TCACTGGAGCAGGGAAGTCC-3′ and 5′-TTCCGCTGTGTAAGCACCTCC-3′; *ABCA1:* 5′-CCCGGCGGAGTAGAAAGG-3′ and 5′-AGGGCGATGCAAACAAAGAC-3′; *GAPDH*: 5′-CTCCCATTCTTCCACCTTTG-3′ and 5′-ATGTAGGCCATGAGGTCCAC-3′. Each set of PCR reactions included water as a negative control and 5 dilutions of the standard. The results showed as the relative expression compared with control levels as described previously [23]. GAPDH was used as the reference gene.

### 2.5. Mutagenesis

To generate the construct of LXR-binding sites mutated *ABCA1* promoter, a construct containing *ABCA1* promoter (pABCA1-LUC) was used as a template and was mutated at -57 and -52 sites with a pair of primer showed following 5′-*CACAGGCTTTGACCAATAGGAACCTCTGCGCTCG*-3′ and 5′-*GTGTCCGAAACTGGTTATCCTTGGAGACGCGAGC*-3′ (mutated nucleotides shown underlined) by PCR as described in site-directed mutagenesis kit. These mutations replace residues that are critical for mediating effects of LXR via its response sequence (*TGACCG* and *TAACCT*).

### 2.6. Luciferase Reporter Gene Assay

Purified pABCA1-LUC was transfected into INS-1 by reagent Lipofectamine2000 (Life Technologies, Gaithersburg, MD, USA). After transfection, cells were maintained in a medium containing HDL or OxLDL for 24 h with or without pre-treatment with LY294002 (10 μM), PD98095 (10 μM), SP600125 (10 μM), or SB203580 (1 μM) to separately inhibit the phosphatidylinositol 3 kinase (PI3K), mitogen-activated protein kinase (MEK), c-Jun N-terminal kinase (JNK), or p38 mitogen-activated protein kinase (p38 MAPK) signaling pathway for 30 min [24]. Then these cells were lysed by buffer, and the *ABCA1* promoter activity was measured according to the manufacturer’s instructions (ToyoInk; Tokyo, Japan).

### 2.7. Chromatin Immunoprecipitation (ChIP) Assay

ChIP assay was performed by the ChIP-IT^TM^ kit (Active Motif, Tokyo, Japan) according to the manufacturer’s instructions. Chromatin was immunoprecipitated with rabbit LXR antibody (Santa Cruz Biotechnology Inc., Dallas, CA, USA) by using control IgG as a negative control. DNA containing the putative LXR-binding sites on rat *ABCA1* promoter was amplified by PCR using forward primer 5′-*GCTTTCTGCTGAGTGACTGAACTAC*-3′ and reverse primer: 5′-*GAATTACTGCTTTTTGCCGCG*-3′ as described previously [17]. PCR was performed using TAKARA PCR thermal cycler MP in the following amplification conditions: 95 °C for 5 min, followed by 36 cycles of 95 °C for 20 s, 59 °C for 30 s, and 72 °C for 30 s. PCR products (229 bp) were detected by 3% agarose gel electrophoresis.

### 2.8. Glucose-Stimulated Insulin Secretion (GSIS)

INS-1 cells were starved in Krebs-Ringer bicarbonate (KRB) buffer containing 120 mM NaCl, 5 mM KCl, 1.1 mM MgCl_2_, 2.5 mM CaCl_2_, 25 mM NaHCO_3_, and 0.1% bovine serum albumin (pH 7.4) for 1 h. After that, cells were rested in KRB buffer containing 3.3 mM glucose for 1 h, and then the medium was changed into KRB buffer containing 3.3 mM glucose or 16.7 mM glucose for 1 h. The supernatant and cell protein was harvested and used for insulin measurement by ELISA kit (Shibayagi, Shibukawa, Japan). The data was normalized by the protein concentration as described previously [17].

### 2.9. Cholesterol Content Assay

As described previously, total cholesterol was measured by a colorimetric assay, which utilizes reagents widely used for the measurement of cholesterol in conjunction with a random-access chemistry analyzer ARCHITECT c8000 [25]. The cholesterol concentration was normalized to protein concentration (µg/µL) each.

### 2.10. Oil Red O Stain

INS-1 cells were seeded on coverslips. After varying treatments, cells were fixed by 4% paraformaldehyde (PFA) for 30 min at room temperature. After washing three timed with phosphate-buffered saline (PBS; pH 7.2), fixed cells were incubated with Oil Red O solution for 15 min. After washing three times with PBS, cells were incubated with hematoxylin solution for 30 s to stain nuclei. After washing three times with PBS, cells were mounted. Pictures were taken by upright microscope (Olympus BX-51/DP-72).

### 2.11. Statistical Analysis

Data were expressed as mean ± SEM. Results were analyzed by one-way ANOVA and Student’s *t* test. *p* < 0.05 was considered as statistically significant. All experiments were performed at least three times.

## 3. Results

### 3.1. Glucose-Stimulated Insulin Secretion (GSIS) Was Impaired by OxLDL-Treatment in INS-1 Cells

**Figure 1:**
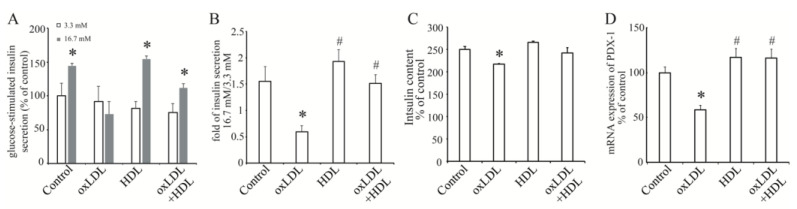
The effect of OxLDL on insulin secretion and synthesis in INS-1 cells. (**A**), glucose-stimulated insulin secretion (GSIS) in INS-1 cells treated with OxLDL at 50 μg/mL, HDL at 10 μg/mL or both. White bar, stimulation with glucose at 3.3 mM; gray bar, stimulation with glucose at 16.7 mM. (**B**), fold of insulin secretion from 3.3 mM glucose to16.7 mM glucose in INS-1 cells. (**C**), the total insulin content in INS-1 cells treated by OxLDL and HDL. (**D**), the effect of OxLDL and HDL on mRNA expression of PDX-1. The ratio is shown as percent of control. Data is presented as the mean ± SEM (*n* = 3) of separate experiments for each group. (**A**), * *p* < 0.05 compared to white bar in each group. (**B**–**D**), * *p* < 0.05 compared to control; # *p* < 0.05 compared to OxLDL.

To analyze the effects of OxLDL on insulin secretion, we used rat insulinoma cell line, an INS-1 cell that has a physiological insulin secretion stimulated by increasing glucose concentration. Our results showed that INS-1 cells treated with OxLDL for 24 h could not secrete more insulin in response to high glucose (Figure 1A). A previous study showed that high-density lipoprotein (HDL) protected insulin secretion from pancreatic beta cells in high-glucose condition [26] and prevented beta cell from apoptosis and oxidative stress induced by OxLDL [27]. In this study, we found that OxLDL with HDL treatment improved impaired insulin secretion, while treatment with only HDL did not enhance this process (Figure 1A). Compared to the fold of insulin release response to high glucose stimulation in control group (1.55 ± 0.28) and HDL group (1.929 ± 0.22), OxLDL remarkably reduced the fold to 0.59 ± 0.12 while treatment with both HDL and OxLDL rescued the fold to 1.51 ± 0.17 (Figure 1B). At the same time, the total insulin content in INS-1 cells with OxLDL was significantly reduced to 86.82 ± 11.11% of control while HDL rescued it to 96.72 ± 4.44% of control (Figure 1C). At the same time, the mRNA expression of *PDX-1*, a critical transcription factor of insulin, was significantly decreased by OxLDL and the addition of HDL protected the basic level of *PDX-1* mRNA expression (Figure 1D). These data demonstrated that OxLDL reduced insulin synthesis and impaired GSIS in INS-1 cells and HDL could protect beta cell function.

### 3.2. OxLDL Induced Cholesterol Accumulation in INS-1 Cells

**Figure 2:**
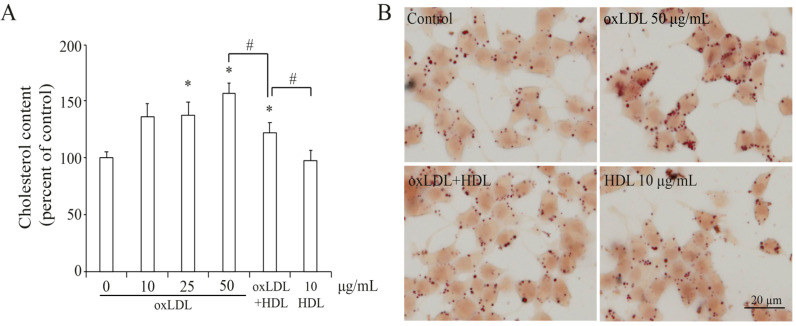
The effect of OxLDL on the cholesterol accumulation in INS-1 cells. (**A**). Percentage of cholesterol content normalized by protein concentration referred to control is presented as the mean ± SEM (*n* = 3) of separate experiments in the graph. * *p* < 0.05 compared to control; # *p* < 0.05 compared to OxLDL + HDL. (**B**) Oil Red O stain of INS-1 cells treated with OxLDL or plus HDL. Bar = 20 µm.

In pancreatic beta cells, impaired insulin secretion could be induced by lipid accumulation [15]. As shown in Figure 2A, treatment with OxLDL for 24 h increased cholesterol content in a dose-dependent manner and the cholesterol accumulation was reduced by the addition of HDL, while only HDL treatment did not affect cholesterol content in INS-1 cells. This was also confirmed by Oil Red O stain that the number and size of lipid droplets were increased by OxLDL, which was reduced by addition of HDL (Figure 2B), confirming that cholesterol accumulation induced by OxLDL contributed to impaired beta cell function.

### 3.3. OxLDL Decreased the ABCA1 Expression in INS-1 Cells

**Figure 3:**
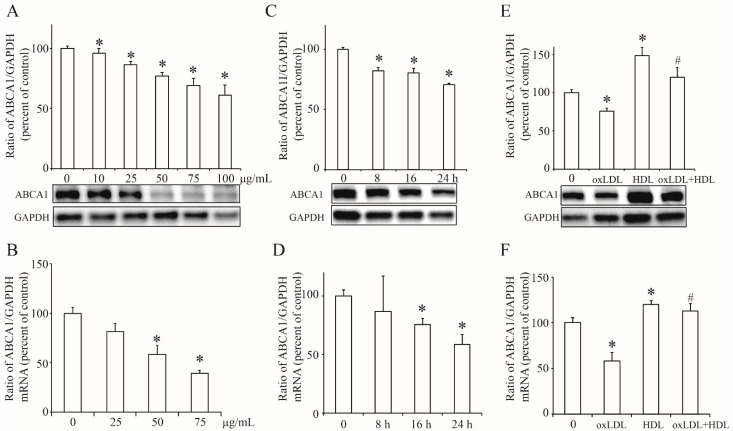
The effect of OxLDL on the expression of ABCA1 in INS-1 cells. (**A**,**B**), the protein and mRNA levels of ABCA1 in INS-1 cells treated with OxLDL at varying concentration (0, 10, 25, 50, 75, 100 µg/mL). (**C**,**D**), the protein and mRNA level of ABCA1 in INS-1 cells with OxLDL at 50 µg/mL for varying time (0, 8, 16, 24 h). (**E**,**F**), the protein and mRNA level of ABCA1 in INS-1 cells with OxLDL at 50 µg/mL or HDL at 10 µg/mL. The ratio of ABCA1 to GAPDH is shown as percent of control. The data is presented as the mean ± SEM (*n* = 3) of separate experiments for each treatment group. * *p* < 0.05 compared to 0; # *p* < 0.05 compared to OxLDL.

As an important transporter of cholesterol, ABCA1 facilitates cholesterol out of cytoplasm to reduce cholesterol content. Then we examined the effect of OxLDL at varying concentration (0~100 µg/mL) on ABCA1 expression and found that OxLDL remarkably decreased ABCA1 expression in a dose-dependent manner in INS-1 cells (Figure 3A). This was confirmed by the results of real-time PCR (Figure 3B). Since OxLDL at 50 µg/mL efficiently decreased the protein and mRNA level of ABCA1, we used this concentration of OxLDL to treat INS-1 cells for varying time (0~24 h) and found it decreased the protein and mRNA expression of ABCA1 expression in time-dependent manner (Figure 3C,D). Furthermore, we found that HDL induced ABCA1 expression in INS-1 cells and prevented the reduction of ABCA1 induced by OxLDL (Figure 3E,F). These results pointed out that OxLDL downregulated ABCA1 expression and might induce cholesterol accumulation in INS-1 cells.

### 3.4. OxLDL Suppressed the Transcription of ABCA1 via LOX-1/MEK/ERK Signaling Pathway in INS-1 Cells

**Figure 4:**
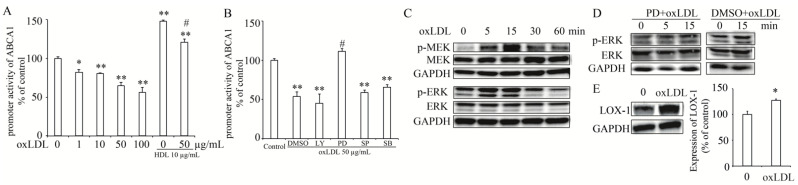
OxLDL suppressed *ABCA1* promoter activity via MEK/ERK pathway in INS-1 cells. (**A**), the effect of OxLDL or HDL on *ABCA1* promoter activity. (**B**), the effects of a PI3K inhibitor LY294002 (LY), a MEK inhibitor PD98059 (PD), a JNK inhibitor SP600125 (SP) or a p38 MAPK inhibitor SB203580 (SB) with or without OxLDL on *ABCA1* promoter activity. Percentage of promoter activity is referred to control. (**C**), phosphorylation of MEK at Ser217/221 and ERK at Thr 202/Tyr 204 in INS-1 cell treated with OxLDL. (**D**), phosphorylation of ERK at Thr 202/Tyr 204 after inhibition of MEK by PD98059 (PD) in INS-1 cells treated with OxLDL. (**E**), the protein expression of LOX-1 in INS-1 cells treated with OxLDL. The ratio is shown as percent of control.Data is presented as the mean ± SEM (*n* = 3) of separate experiments for each treatment group. * *p* < 0.05, ** *p* < 0.01 compared to 0 or control; # *p* < 0.05 compared to OxLDL at 50 µg/mL or DMSO plus OxLDL.

To further investigate the effect of OxLDL on the transcription of *ABCA1* gene, we used a constructed luciferase reporter plasmid containing the promoter region of *ABCA1*. As shown in Figure 4A, OxLDL suppressed the promoter activity of *ABCA1* in a dose-dependent manner while it was enhanced by addition of HDL. To identify the signaling pathway involved in OxLDL-suppressed *ABCA1* transcription, we used several inhibitors LY (LY294002, 10 μM), PD (PD98095, 10 μM), SP (SP600125, 10 μM) or SB (SB203580, 1 μM) to separately inhibit the PI3K, MEK, JNK, or p38 MAPK signaling pathway. As shown in Figure 4B, only inhibition of the MEK pathway by PD cancelled the effect of OxLDL on *ABCA1* promoter activity (Figure 4B), suggesting that OxLDL may regulate ABCA1 expression via MEK signaling pathway. Further, the activation of MEK/ERK (extracellular signal-regulated kinase) was stimulated by treatment with OxLDL for a different time (0~60 min) in INS-1 cells (Figure 4C). Ser217/221-phosphorylated MEK was activated from 5 min and peaked at 15 min after OxLDL treatment while Thr202/Tyr204 phosphorylation of ERK was detected from 5 min to 15 min (Figure 4C). Next, inhibition of MEK by PD98095 cancelled the activation of ERK induced by OxLDL (Figure 4D). Lectin-like OxLDL receptor-1 (LOX-1) was identified as a major receptor for the uptake of OxLDL [9] and activation of ERK pathway was shown to be mediated by LOX-1 [28]. In our study, the expression of LOX-1 was significantly increased in INS-1 cells (Figure 4E), suggesting that OxLDL suppressed *ABCA1* transcription via LOX-1/MEK/ERK pathway in INS-1 cells.

### 3.5. LXR Involved in the Process of OxLDL-Suppressed ABCA1 Expression

**Figure 5:**
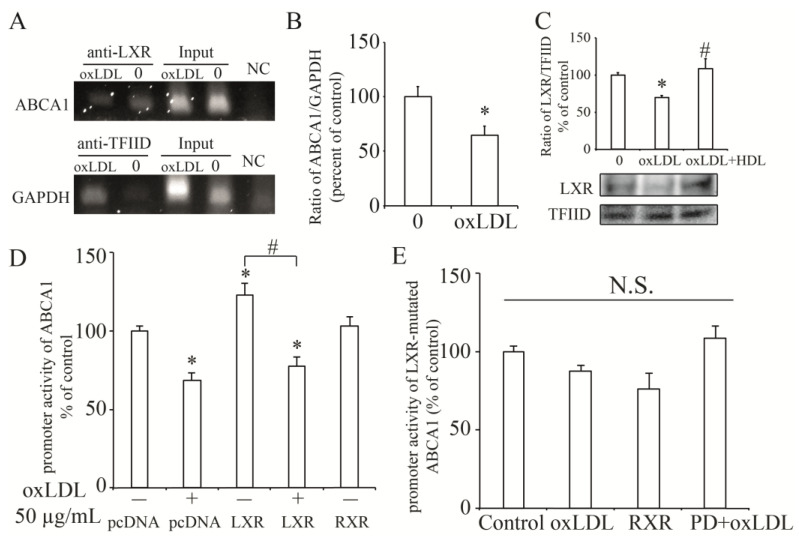
Roles of transcription factor, LXR in OxLDL-suppressed ABCA1 transcription. (**A**), the direct binding of LXR to *ABCA1* promoter. LXR specifically immunoprecipitated *ABCA1* chromatin (anti-LXR) while TFIID specifically immunoprecipitated *GAPDH* chromatin (anti-TFIID) in INS-1 cells by ChIP assay. No ChIP was detected when the chromatin was immunoprecipitated with negative control IgGs (NC). Input was shown as a positive control. There is no band detected when the templates were amplified by negative primer. (**B**), the effect of OxLDL on LXR-binding to *ABCA1* promoter by ChIP-real time PCR. (**C**), the nuclear abundance of LXR protein in INS-1 cells treated with OxLDL or OxLDL plus HDL. (**D**), the role of LXR in *ABCA1* promoter activity with OxLDL treatment. (**E**), the effect of OxLDL or inhibition of MEK on the LXR-binding sites (*G*ATAG*T* to *A*ATAG*G*) mutated *ABCA1* promoter activity. * *p* < 0.05 compared to control (0, pcDNA). # *p* < 0.05 compared to OxLDL at 50 µg/mL (**C**). # *p* < 0.05 compared to LXR group (**D**). N.S., No significance.

Next, we tried to search a transcription factor of the *ABCA1* gene, which is downstream of the MEK/ERK pathway. Previous studies proved that transcription factor, liver X receptor (LXR), is able to regulate *ABCA1* transcription combined with retinoid X receptor (RXR) [18,29] and serves as a downstream of MEK/ERK pathway in INS-1 cells [17]. In the present study, we used chromatin immunoprecipitation (ChIP) assay to confirm that LXR could directly bind to the *ABCA1* promoter region in INS-1 cells (Figure 5A) and ChIP-real time PCR showed that this binding was significantly reduced by the treatment with OxLDL (Figure 5B). Moreover, OxLDL remarkably decreased nuclear expression of LXR while addition of HDL rescued this reduction (Figure 5C), indicating that OxLDL inhibited *ABCA1* transcription by LXR. When we overexpressed LXR and its cotransfector RXR in INS-1 cells, *ABCA1* promoter activity was enhanced, while only overexpression of RXR had no effect on *ABCA1* promoter activity (Figure 5D). However, the LXR-enhanced *ABCA1* promoter activity was suppressed by treatment of OxLDL. Further, when we mutated the LXR-binding sites (GATAGT to AATAGG) in *ABCA1* promoter region, treatment with OxLDL, PD plus OxLDL, or overexpression of RXR had no stimulatory effect on this mutated promoter activity (Figure 5E). These findings supported the idea that OxLDL inhibited ABCA1 transcription by LXR in INS-1 cells.

## 4. Discussion

In the present study, we investigated the effect of OxLDL on dysfunction of pancreatic beta cells in detail. Our results showed that OxLDL inhibited GSIS, insulin content, and PDX-1 expression by cholesterol accumulation, which might be induced by suppressing pancreatic ABCA1 via MEK/ERK/LXR pathway in the pancreatic beta cell line, INS-1 cells.

OxLDL induces cholesterol accumulation, which is caused by down-regulated ABCA1 expression via MEK/ERK/LXR pathway. PDX-1 expression is reduced by OxLDL. As a result, insulin synthesis and glucose-stimulated insulin secretion (GSIS) is impaired. C, cholesterol; P, phosphorylation of MEK or EKR.

OxLDL was found to be linked to pathologic conditions related to cardiovascular diseases (CVDs), including diabetes mellitus, obesity, and metabolic syndrome. It has been shown that high levels of OxLDL, glucose, insulin resistance, and free fatty acid with low levels of HDL cholesterol result in the upregulation of scavenger receptor, thereby contributing to T2D and related atherosclerosis [30]. A clinical study on patients with metabolic syndrome followed up for 20 years showed that incidence of hyperglycemia, hypertriglyceridemia, and abdominal obesity increased, closely associating with a higher OxLDL level [21]. This study is confirmed by a following study that OxLDL exhibited a positive relationship with T2D in 7 years of follow-up [3], pointing out the harmful effect of OxLDL on inducing T2D. In vivo experiments demonstrated that blocking of OxLDL by its antibody improves insulin sensitivity [7]. In vitro, proinsulin synthesis and insulin secretion were suppressed by OxLDL [4] and cholesterol accumulation contributed to impaired insulin secretion from beta cells in the condition of high glucose [31]. In this study, we found OxLDL suppressed insulin synthesis and impaired GSIS in INS-1 cells and these effects were prevented by addition of HDL, confirming with previous research that HDL protected beta cells from apoptosis and oxidative stress induced by OxLDL [27]. Clinical study showed that HDL significantly improved glucose tolerance and increased plasma insulin by stimulating ABCA1 expression in T2D patients [32]. Moreover, in high-glucose condition, HDL protected insulin secretion and this effect was a ABCA1-dependent manner in pancreatic beta cells [26]. Consistently in our study, HDL stimulated ABCA1 expression and transcription in INS-1 cells. These evidences indicate the important role of ABCA1 in protecting insulin secretion in pancreatic beta cells.

Accumulation of cholesterol content in pancreatic islets, either in *ob*/*ob* mice or in cholesterol-overloaded beta cell line, impairs insulin release response to glucose [33]. Consistently, high levels of cholesterol content in pancreatic islets and reduction of insulin secretion are detected in mice lacking beta cell ABCA1 [15,16], suggesting that cholesterol accumulation induced by absence of ABCA1 may induce dysfunction of beta cells. A recent study has demonstrated that in ApoE-knockout mice with nicotine-pretreatment, enhancement of OxLDL uptake by macrophages aggravated atherosclerotic plaque formation through downregulation of ABCA1 [11]. In this study, we found cholesterol content was increased by OxLDL while insulin content and GSIS were decreased by OxLDL in INS-1 cells. In agreement with the previous report, we found OxLDL remarkably suppressed ABCA1 expression of INS-1 cells in a dose-dependent and time-dependent manner. A previous study indicated that pancreatic ABCA1 played an important role in glucose homeostasis regulated by rosiglitazone [15]. Additionally, our previous studies showed that an analogue of the glucagon-like peptide 1 (GLP-1) and insulin-like growth factor-1 (IGF-1) could stimulate the expression of ABCA1 and enhance insulin secretion in pancreatic beta cells while angiotensin II and TNF-alpha have the opposite effects on it [17,25,34,35,36]. These results suggested that decreased cholesterol content by upregulation of ABCA1 with several treatments could protect pancreatic beta cells from dysfunction. ABCA1 is firstly identified as a mutated molecule in Tangier Disease, with deficiency of high-density lipoprotein (HDL), accumulation of cholesterol in many cases, and glucose intolerance developing into diabetes [37]. A clinical report showed that four Japanese patients with Tangier Disease and T2D exhibited lower value of the insulinogenic index insulin compared to non-diabetic TD patients [38], indicating that cholesterol content and ABCA1 may be critical for insulin secretion.

Uptake of OxLDL by its receptors plays an important role in increasing oxidative stress and promoting foam cells’ formation. Previous evidence showed that uptake of OxLDL was increased by upregulation of the scavenger receptor CD36 [10]. However, increasing the uptake of OxLDL by over-expressing CD36 did not enhance the lipotoxicity but induced more apoptosis of INS-1 cells [39], showing that there might be other receptors involved in OxLDL impaired GSIS. Previously, blocking LOX-1 by its antibody attenuated OxLDL-stimulated ER stress in endothelial cells [40]. Consistently, we found that the expression LOX-1 was elevated by the treatment of OxLDL, and HDL rescued the effect of OxLDL on dysfunction of INS-1 cells.

One of the goals in this study was to study the signaling pathway activation involved in OxLDL-mediated *ABCA1* gene expression. Present results showed that OxLDL suppressed *ABCA1* promoter activity and only inhibition of MEK by its specific inhibitor, PD98059 altered this effect, suggesting that MEK is required in OxLDL-regulated *ABCA1* transcription. Moreover, phosphorylation of MEK at Ser217/221 sites was activated by the treatment of OxLDL. Activation of MEK is able to phosphorylate mitogen-activated protein kinases (MAPKs), which are a family of serine/threonine protein kinases to modulate cell growth and differentiation [41]. ERK, one member of the MAPK family, contains p44 (ERK1) and p42 (ERK2) and is activated by phosphorylation of threonine and tyrosine residues by MEK [42]. ERK signaling is able to be stimulated to regulate function of vascular smooth muscle cells via OxLDL receptors [28]. In our study, MEK/ERK pathway was activated by the treatment of OxLDL, and this was cancelled by inhibition of MEK, which may contribute to the dysfunction of pancreatic beta cells. In our previous study, the MEK/ERK pathway activated by angiotensin II type 1 receptor (AT1) was shown to regulate pancreatic *ABCA1* promoter activity [17]. A recent report showed that AT1 could be activated by OxLDL via binding to LOX-1 [43], pointing out that OxLDL may stimulate MEK/ERK phosphorylation. Consistently in present study, our results showed that activation of MEK/ERK mediated OxLDL-inhibited *ABCA1* transcription activity in INS-1 cells.

Several studies demonstrated that inhibition of ERK1/2 and activation of liver X receptor (LXR) synergistically reduced atherosclerotic lesions in ApoE-deficient mice [44] and activation of MEK/ERK modulates LXR-dependent ABCA1 expression [45]. LXR is expressed in a variety of tissues including the pancreas and is well-known in regulating lipid homeostasis and metabolism, facilitating cholesterol efflux from cells, and protecting the cells from lipid toxicity [46]. LXR/RXR heterodimers, upon binding to LXR response elements (LXREs), lead to the transcription of target genes, including *ABCA1* [47]. Then, the DR4 site in the *ABCA1* promoter is able to be activated by LXR, which is critical to the upregulation of ABCA1 and cholesterol efflux [29]. LXR-mediated *ABCA1* gene expression plays an important role in hepatic HDL biogenesis and macrophage cholesterol efflux [48]. Particularly in pancreatic beta cells, our results indicated that OxLDL decreased *ABCA1* transcription by reducing the nuclear content of LXR in INS-1 cells and this reduction was prevented by addition of HDL. As shown in previous reports, transcription factor peroxisome proliferator activator receptors (PPARs) was also downstream of ERK pathway [49] and activated pancreatic ABCA1 expression [50]. Recently, RXR has been proposed as one of the therapeutic targets for treating obesity-related metabolic disorder. A novel small molecule agonist of RXR, UAB126 could prevent high-fat diet induced glucose intolerance [51] and retinoic acid isomer; 9ciRA balanced cholesterol homeostasis by regulating ABCA1 expression [52]. Further experiments will be needed to clarify a network regulation of several transcriptional factors including LXR, RXR, and PPARs in OxLDL-suppressed pancreatic ABCA1.

In summary, OxLDL suppresses the expression of pancreatic ABCA1 via MEK/ERK/LXR pathway, leading to cholesterol accumulation and the inability of INS-1 cells to secrete insulin. These findings provide a basic molecular knowledge in the effect of lipotoxicity on beta cell function, contributing to the understanding the pathological mechanism of T2D.

## Data Availability

The datasets used and/or analysed during the current study are available from the corresponding author upon reasonable request.

## Abbreviations

OxLDL, oxidized low-density lipoprotein; HDL, high-density lipoprotein; ER, endoplasmic reticulum; LXR, liver X receptor; RXR, retinoid X receptor; LOX-1, lectin-type oxidized LDL receptor 1; ChIP, chromatin immunoprecipitation; GSIS, glucose-stimulated insulin secretion; PI3K, phosphatidylinositol 3 kinase; MEK, mitogen-activated protein kinase kinase; JNK, c-Jun N-terminal kinase; p38 MAPK, p38 mitogen-activated protein kinases; ERK, extracellular signal-regulated kinase; IGF-1, insulin-like growth factor-1; TD, Tangier disease; CVDs, cardiovascular diseases.
